# The effects of dimethyl fumarate and fingolimod on T-cell lymphocyte proliferation in patients with multiple sclerosis

**DOI:** 10.1007/s11845-021-02913-8

**Published:** 2022-01-13

**Authors:** Audrey Reynolds, Maria Gaughan, Dean Holden, Vyanka Redenbaugh, Jean Dunne, Janice Redmond, Niall Conlon

**Affiliations:** 1 Department of Neurology, St James’s Hospital, Dublin 8, Ireland; 2 Department of Immunology, St James’s Hospital, Dublin 8, Ireland

**Keywords:** Dimethyl fumarate, Fingolimod, Lymphocyte proliferation, Multiple sclerosis

## Abstract

**Objective:**

The disease-modifying therapies (DMT), dimethyl fumarate (DMF) and fingolimod (FTY) improve the outcomes in multiple sclerosis (MS) by reducing relapses and numbers and volume of lesions. They mediate their effects through reduction of immune reactivation, which may potentially lead to lymphopaenia and increased risk of infections. Previous studies have examined the effects of these therapies on lymphocyte subsets; however, the in vivo effects on circulating lymphocyte proliferation require further elucidation. The aim of this study was to determine the effects of DMF and FTY on T-cell proliferation in patients with MS.

**Method:**

We examined T-cell lymphocyte proliferation and lymphocyte subsets in ten patients (five on DMF, five on FTY) before starting DMT and again 4 to 11 months after being maintained on DMT.

**Results:**

In the FTY-treated group, the mean percentage proliferation was significantly lower using both assays (PHA assay mean percentage change − 51.2 ± 25.97, *p* < 0.05; anti-CD3/CD28 assay mean percentage change − 39.74 ± 27.85, *p* < 0.05). There was no statistical difference in T-cell lymphocyte proliferation in the DMF-treated group for either assay (PHA, *p* = 0.316; anti-CD3/CD28, *p* = 0.373).

**Conclusions:**

This pilot study suggests that the T-lymphocytes of patients on FTY have an abnormal proliferation response as well as being reduced in the circulation.

## Introduction

Multiple sclerosis (MS) is a chronic neuroinflammatory disorder associated with a typically relapsing remitting course of neurological deficits and the accumulation of physical and cognitive disability over time [1]. Fingolimod (FTY) and dimethyl fumarate (DMF) are medications used in the treatment of relapsing remitting multiple sclerosis which exert their effects through modification of the immune system. FTY prevents the egress of lymphocytes from lymph nodes, reducing the amount available in circulation [2]. DMF has immune mediating properties which result in selective depletion of CD8 over CD4 cells [3]. Both may result in lymphopaenia and increased risk of infections [4]. FTY in particular has been associated with a number of cases of cryptococcal meningitis [5]. Previous studies have examined the effects of these therapies on lymphocyte subsets, showing that FTY and DMF target different lymphocyte compartments [6]. Lymphocyte proliferation assays are an investigative tool to assess the function of lymphocytes and are used clinically in the diagnosis of immunodeficiencies and other immune system disorders [7].

Our pilot study aimed to measure lymphocyte number and T-cell lymphocyte proliferative capacity using a new fully accredited lymphocyte proliferation assay in a small cohort of MS patients who were starting either FTY or DMF.

## Methods

The study was approved by the hospital ethical committee. Written informed consent was obtained from patients prior to blood draw. Ten patients with a diagnosis of relapsing remitting multiple sclerosis were recruited prior to starting oral disease modifying therapy. Treatment was not blinded and based on clinical decision and patient preference. Five patients were about to initiate FTY therapy, and five were about to initiate DMF. Of the DMF group, one patient had been on no prior disease-modifying therapies (DMT), two on interferon previously and two had been on glatiramer acetate previously. Of those starting therapy with FTY, one had been on no prior medications, two had previously been on glatiramer acetate, one on interferon only previously and one patient had previously trialled both interferon and dimethyl fumarate. Demographic data is listed in Table 1. Peripheral blood mononuclear cell (PBMCs) samples for the T-cell proliferation assay were collected in BD Vacutainer® CPT™ tubes, and samples for lymphocyte subset enumeration (whole blood in EDTA tubes) were taken before initiation of therapy and on average 6 months (range 4 to 11 months) after being maintained on therapy.

**Table 1:** Demographic data. There were five patients in the DMF-treated group and five patients in the FTY-treated group

Demographics	DMT	
	FTY	DMF
Age	38.2 ± 9.7	46 ± 16.2
*Sex*		
Male	2	3
Female	3	2
EDSS	3.4 ± 1.8	4.1 ± 1.3
Years since diagnosis	6.8 ± 5.5	19 ± 6.4

Fresh PBMCs for T-cell proliferation were isolated from the CPT tubes by centrifugation. The isolated PBMCs were processed and incubated under standard cell-culture conditions. T-cell proliferation was stimulated using the non-specific mitogen PHA (phytohaemagglutinin) and the specific stimulator anti-CD3/CD28. Click-iT® EdU (5-ethynyl-2′-deoxyuridine) was used to identify proliferation; it uses a nucleoside analog to thymidine (EdU) which is incorporated into DNA during synthesis and this allows the identification of proliferation by using click reaction where the EdU is labelled with a fluorescent tag (AF488). T-cells were assessed for evidence of T-cell proliferation 96 h post-stimulation by surface staining with anti-CD45 and anti-CD3 to identify T-cells and a click chemistry reaction to label the incorporated EdU with AF488. Results are expressed as percentage lymphocyte stimulated, which represents the percentage of the total which incorporate EdU dye indicating proliferation. Samples were analysed on a BD FACSCanto II using BD FACSDiva software. A normal healthy control was also included within each assay. This method was designed and validated in-house (ISO 15189 accredited) and is in routine use for clinical testing. Lymphocyte subsets were enumerated using BD Multitest™ CD45/CD3/CD4/CD8 antibody cocktail and BD Trucount™ tubes — this is an IVD method which is ISO 15189 accredited. Paired-sample *t* test was conducted using GraphPad Prism version 8.1.

## Results

**Fig. 1:**
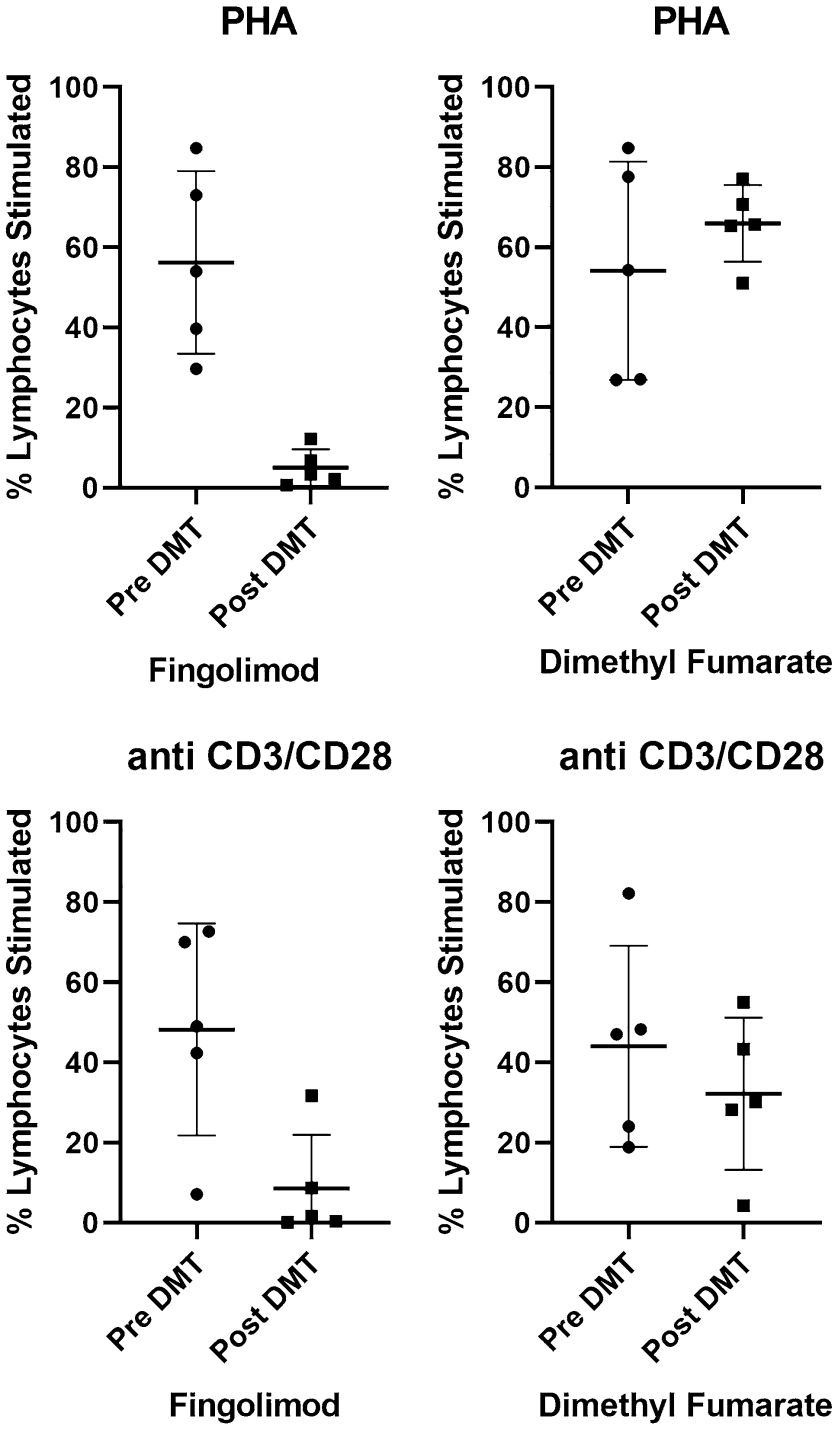
T-cell lymphocyte proliferation. Lymphocyte proliferation assays showing percentage of T-cell lymphocytes stimulated using mitogens PHA and anti-CD3/CD-28 before and after starting treatment. Treatment with FTY caused significant reductions in percentage of T-lymphocytes stimulated using both mitogens (PHA, *p* = 0.016; anti-CD3/C28, *p* = 0.033). Treatment with DMF did not cause significant change in percentage T-lymphocyte stimulated (PHA, *p* = 0.3155; anti-CD3/CD28, *p* = 0.3731). PHA, phytohaemagglutinin; FTY, fingolimod; DMF, dimethyl fumarate; DMT, disease-modifying therapies

All patients treated with FTY developed lymphopaenia (pretreatment mean 1.6 ± 0.34 10^9^/L; post-treatment mean 0.34 ± 0.05 10^9^/L) while one patient in the DMF-treated group developed lymphopaenia (pretreatment mean 2.58 ± 1.47 10^9^/L; post-treatment mean 2.275 ± 1.12 10^9^/L). DMF did not cause significant changes in CD4 count (pretreatment mean 1028 ± 506 10^6^/L; post-treatment mean 671 ± 507 10^6^/L; *p* = 0.069) or CD8 count (pretreatment average 603 ± 425 10^6^/L; post-treatment average 394 ± 465 10^6^/L; *p* = 0.1). FTY reduced CD4 counts significantly (pretreatment average 780 ± 184 10^6^/L; post-treatment average 57 ± 87 10^6^/L; *p* = 0.002) without significant changes in CD8 counts (pretreatment average 372 ± 275 10^6^/L; post-treatment average 115.6 ± 77 10^6^/L; *p* = 0.05) (Table 2). In the FTY-treated group, the mean percentage proliferation was significantly lower than pretreatment using both mitogens (PHA assay mean percentage change − 51.2 ± 25.97, *p* = 0.0116; anti-CD3/CD28 assay mean percentage change − 39.74 ± 27.85, *p* = 0.0332). There was no statistical difference in T-lymphocyte proliferation in the DMT-treated group for either assay (PHA, *p* = 0.316; anti-CD3/CD28, *p* = 0.373) (Fig. 1).

**Table 2:** Absolute lymphocyte count and lymphocyte subsets before and after starting DMT. FTY but not DMF caused significant lymphopaenia. Treatment with FTY caused significant reductions in CD4 count but not CD8 count. DMF treatment did not significantly affect CD4 or CD8 count

Count	Pre-DMT		Post-DMT		Paired *t* test two tailed significance
	Mean	SD	Mean	SD	*p* value
FTY lymphocyte count (10^9^/L)	1.60	0.34	0.34	0.05	0.001
DMF lymphocyte count (10^9^/L)	2.58	1.47	2.28	1.12	0.683
*Lymphocyte subsets*					
FTY CD4 (10^6^/L)	780	184	57	87	0.002
DMF CD4 (10^6^/L)	1028	506	671	507	0.069
FTY CD8 (10^6^/L)	372	275	116	77	0.050
DMF CD8 (10^6^/L)	603	425	394	465	0.100

## Discussion

In this pilot study, we found substantive differences between FTY and DMF with respect to both lymphocyte number and T-cell proliferative activity. All of the patients treated with FTY developed lymphopaenia, as expected based on prevention of egress of lymphocytes from lymph nodes [2]. None of the DMF-treated patients in our group developed significant lymphopaenia.

Peripheral blood T-lymphocytes from FTY-treated MS patients have abnormal proliferation, but proliferative capacity was normal in DMF-treated patients. This may imply a more significant immunosuppressive effect than previously described. Similar to previous studies [6], FTY-treated patients developed severe reductions in CD4 count and a reversal of the CD4/CD8 ratio. This in conjunction with the abnormalities in lymphocyte proliferation may clinically contribute to increased risk of infections. Disorders which affect lymphocyte proliferation, such as combined immunodeficiency states, are associated with severe immunodeficiency and opportunistic infection risk [8]. FTY in particular can be associated with cryptococcal meningitis [5], an infection described in other conditions which affect CD4 count such as HIV [9].

In this study, T-lymphocyte proliferation was not affected by treatment with DMF. Other studies have found that DMF can influence lymphocyte proliferative ability in vitro [10]. Whether lymphocyte proliferation is abnormal in the cohort of those treated with DMF who develop lymphopaenia needs to be further elucidated.

Our study has a number of limitations. It contained a small number of patients, five in each treatment group. Patients were unrandomised, having started either medication based on clinical decision and patient preference. Due to lack of randomisation, patients in the DMF treatment were slightly older than the FTY-treated group. Immunosenescence can result in lower rates of lymphocyte proliferation as patients age [11]; however, as the DMF group was older than the FTY group, this does not explain the lower rates of lymphocyte proliferation in the FTY group.

This was a small non-randomised comparative study that will require replication in larger cohorts. The large reduction in lymphocyte proliferative capacity in patients treated with FTY compared to DMF merits further analysis.

## Conclusion

The pilot study has found that FTY, but not DMF, caused substantial reductions in T-cell lymphocyte proliferative ability. Further investigations to fully characterise these changes in lymphocyte function are required.
